# Quality of life assessment in interstitial lung diseases:a comparison of the disease-specific K-BILD with the generic EQ-5D-5L

**DOI:** 10.1186/s12931-018-0808-x

**Published:** 2018-05-25

**Authors:** Boglárka Lilla Szentes, Michael Kreuter, Thomas Bahmer, Surinder S. Birring, Martin Claussen, Julia Waelscher, Reiner Leidl, Larissa Schwarzkopf

**Affiliations:** 1 Helmholtz Zentrum München – German Research Center for Environmental Health (GmbH), Institute of Health Economics and Health Care Management, Comprehensive Pneumology Center Munich (CPC-M), Member of the German Center for Lung Research (DZL), Ingolstaedter Landstrasse 1, 85764 Neuherberg, Germany; 2 Center for Interstitial and Rare Lung Diseases Pneumology and Respiratory Critical Care Medicine, Thoraxklinik University of Heidelberg, Translational Lung Research Center Heidelberg (TLRC), Member of the German Center for Lung Research (DZL), Roentgenstrasse, 169126 Heidelberg, Germany; 3 LungenClinic Grosshansdorf GmbH Pneumology, Member of the Airway Research Center North (ARCN), German Center for Lung Research (DZL), Wöhrendamm, 80 22927 Großhansdorf, Germany; 4 Centre of Human & Aerospace Physiological Sciences, School of Basic & Medical Biosciences Faculty of Life Sciences & Medicine, King’s College London, London, WC2R 2LS UK; 5 Department of Respiratory Medicine, King’s College Hospital, Denmark Hill, Brixton, London, SE5 9RS UK; 6 Munich Center of Health Sciences, Ludwig-Maximilians-Universität München, Ludwigstr. 28, 80539 Munich, RG Germany

**Keywords:** ILD, Health-related quality of life, K-BILD, EQ-5D-5L, Comorbidities

## Abstract

**Background:**

Patients with interstitial lung diseases (ILD) have impaired health-related quality of life (HRQL). Little is known about the applicability of the disease-specific King’s Brief Interstitial Lung Disease questionnaire (K-BILD) and the generic EQ-5D-5L in a German setting.

**Methods:**

We assessed disease-specific (K-BILD) and generic HRQL (EQ-5D experience based value set (EBVS) and Visual Analog Scale (VAS)) in 229 patients with different ILD subtypes in a longitudinal observational study (HILDA). Additionally, we assessed the correlation of the HRQL measures with lung function and comorbidities. In a linear regression model, we investigated predictors (including age, sex, ILD subtype, FVC percentage of predicted value (FVC%pred), DLCO percentage of predicted value, and comorbidities).

**Results:**

Among the 229 patients mean age was 63.2 (Standard deviation (SD): 12.9), 67.3% male, 24.0% had idiopathic pulmonary fibrosis, and 22.3% sarcoidosis. Means scores were as follows for EQ-5D EBVS 0.66(SD 0.17), VAS 61.4 (SD 19.1) and K-BILD Total 53.6 (SD 13.8). K-BILD had good construct validity (high correlation with EQ-5D EBVS (0.71)) and good internal consistency (Cronbach’s alpha 0.89). Moreover, all HRQL measures were highly accepted by patients including low missing items and there were no ceiling or floor effects. A higher FVC % pred was associated with higher HRQL in all measures meanwhile comorbidities had a negative influence on HRQL.

**Conclusions:**

K-BILD and EQ-5D had similar HRQL trends and were associated similarly to the same disease-related factors in Germany. Our data supports the use of K-BILD in clinical practice in Germany, since it captures disease specific effects of ILD. Additionally, the use of the EQ-5D-5L could provide comparison to different disease areas and give an overview about the position of ILD patients in comparison to general population.

**Electronic supplementary material:**

The online version of this article (10.1186/s12931-018-0808-x) contains supplementary material, which is available to authorized users.

## Background

Interstitial lung diseases (ILDs) comprise more than 200 rare diseases, which are characterized by varying degrees of inflammation and fibrosis of the lung, and are associated with serious quality of life impairments in affected people [1–5]. There were attempts to quantify the health-related quality of life (HRQL), however most previous analyses focused on the most prevalent forms of ILDs (i.e. Idiopathic Pulmonary Fibrosis (IPF), Sarcoidosis) e.g. Kreuter et al. provide data about the German IPF population [6] or did not apply ILD-specific assessment tools [6–13]. Instead, among others, questionnaires originally designed for patients with Chronic Obstructive Pulmonary Disease (COPD) were tested in ILD-populations: e.g. the COPD Assessment Test [7] and the St George’s Respiratory Questionnaire (SGRQ) [5, 8]. The suitability of these questionnaires to reflect ILD-specific aspects of HRQL remains up to discussion. Moreover, given the heterogeneous clinical course of ILDs, a transferability of HRQL findings among patients with IPF or sarcoidosis to other ILD subtypes is a highly sensitive issue.

Keeping these drawbacks in mind, all studies cited suggest impaired HRQL in ILD patients but comprehensive analyses of HRQL in ILDs accounting for many different subtypes and focusing on disease-specific questionnaires are sparse.

Recently the King’s Brief Interstitial Lung Disease Questionnaire (K-BILD) [9] has been proposed as the first and so far only ILD-specific HRQL assessment tool. The K-BILD is a validated [10] and clinically oriented HRQL tool [11]. Evidence shows that K-BILD is a suitable HRQL measure in different countries; e.g. in UK [9] and in Italy, France, Sweden and the Netherlands as shown by Wapenaar et al. [10] However, until now there is no study using K-BILD in a German setting beyond Kreuter et al. that have translated and validated the questionnaire [12] in 2016.

To compare the disease burden of ILD patients with the general population or with patients suffering from different diseases, the use of a generic HRQL instrument is recommended, since generic questionnaires measure overall HRQL and not just disease-specific primarily symptom-driven aspects, which would not apply for every group [14]. The EuroQol group developed the generic EuroQol five dimensional 5-Level (EQ-5D-5L) questionnaire, which is the improved version of the well-known and well-established 3-level version; EQ-5D-3L [13]. Thus, we assume that the 5L version would provide a good insight in the generic HRQL in the ILD patients and allows the comparison with disease-specific measures. The use of the EQ-5D-5L in lung disease patients is spare so far [10, 15, 16], and there is no validation in the ILD disease-area yet.

Therefore, in the first step we aimed to investigate the suitability of the K-BILD in Germany to measure ILD-specific HRQL. In the second step we aimed to measure psychometric values of the EQ-5D-5L compared to the K-BILD and thereby contribute to a validation of the generic HRQL measure in the disease group ILD. Furthermore, we want to give first insights into HRQL of ILD patients and its predictors in a German tertiary care setting.

## Methods

### Study population and data collection

Data is derived from the ongoing HILDA (**H**ealth Care in **ILD A**mbulance Visitors) study. This observational study addresses outpatients diagnosed with any ILD subtype who presented to the outpatient practices of two large German tertiary care centers for ILD in Germany (Thoraxklinik Heidelberg, LungenClinic Großhansdorf). Heidelberg is a city in south-west Germany whereas Grosshansdorf is in the Northern part. Participants were recruited sequentially over a period of six months starting in November 2016. The local Ethics Committees of Heidelberg and Luebeck approved the study (reference number S-200/2013, and AZ: 16-192, respectively). Participants provided written informed consent.

Individuals who were 18 years of age and older, with ILD confirmed by the ILD boards of the respective centers, with an expected survival time of more than 12 months and with sufficient knowledge of the German language were eligible for the HILDA-study. Participants were grouped to one of the following ILD subtypes: ‘IPF’, sarcoidosis, Hypersensitivity Pneumonitis (HP), other Idiopathic Interstitial Pneumonias (than IPF) (‘other IIP’), and other ILDs based on the differential diagnosis of the treating clinician. ‘Other IIP’ accounts for idiopathic interstitial pneumonia, non-specific interstitial pneumonia, desquamative interstitial pneumonia, cryptogenic organizing pneumonia, lymphocytic interstitial pneumonia, respiratory bronchiolitis-associated interstitial lung disese, pleuropulmonary fibroelastosis, and acute interstitial pneumonia, while the ‘other’ group includes every other subtype not listed above.

### HRQL assessment

The patients’ self-reported HRQL was assessed at time of inclusion (at baseline) into the HILDA registry as part of their regular ambulance visits using EQ-5D-5L (generic HRQL) [17] and K-BILD [9] (disease-specific HRQL).

- EQ-5D-5L

The generic EQ-5D-5L consists of two parts, the descriptive system and a Visual Analogue Scale. The descriptive system addresses five different dimensions (‘mobility’ , ‘self-care’ , ‘usual activities’, ‘pain/discomfort’, and ‘anxiety/depression’), each with a five point Likert-Scale. The answering pattern can be transferred to a utility between 0 and 1 (the higher the better) by distinct (nation-specific) scoring algorithms [18–21]. We chose the Germany-specific experience-based value set (EQ-5D EBVS) from Leidl et al. [18] for calculation of values. The Visual Analog Scale (VAS) allows valuing current health on a thermometer scale between 0 and 100, with higher values indicating better health.

b) K-BILD

K-BILD measures health impairments induced by ILD. The questionnaire covers 15 questions spread out in three domains (‘breathlessness and activity’, ‘chest symptoms’ and ‘psychological impact’) via a seven point Likert Scale. The total (cross-domain) score and domain-specific subscores range from zero to 100 with higher values indicating better health. Scores can be calculated through a predefined, not a patient-reported scoring algorithm, which is provided by the authors upon request [9].

### Assessment of covariables

To reflect potential impact factors on HRQL we accounted for comorbidity burden, clinical aspects and the patients’ sociodemographic background.

Comorbid conditions were derived from patients’ history and medical records. We considered the following comorbid conditions based on previous evidence on their ILD-relevance: pulmonary hypertension, arterial hypertension, coronary heart disease, congestive heart failure, other cardiovascular disease, diabetes mellitus, emphysema/COPD, lung cancer, depression, gastroesophageal reflux disease, renal failure, obstructive sleep apnea, thromboembolism, and malignant tumors other than lung cancer [22–24]. In addition, physicians were allowed to list up to three relevant comorbid conditions not included in the pre-selection. Comorbidity burden was operationalized as sum of all documented conditions, therefore ranging between zero and 17.

As clinical routine, we measured forced vital capacity percent predicted (FVC % pred), and diffusing capacity of the lungs for carbon monoxide percent predicted (DLCO % pred) as functional parameters.

We assessed further basic characteristics by questionnaire-based self-reports of the patients.

### Statistical analysis

For our analyses, only patients with complete information on HRQL and on all covariables relevant for regression analyses were included (complete case analysis). The data from the HRQL questionnaire were considered complete if the total score could be calculated. To avoid selection bias, we compared patient characteristics of those with incomplete and complete questionnaires before finally excluding any patients from further analyses. Subsequently, we assessed floor and ceiling effects; defined as > 15% of the participants achieving the best/worst HRQL score [25]. Besides, correlations between the HRQL measures, lung function parameters and the comorbidity sum score were quantified by Spearman’s rank coefficient to examine associations. We considered correlations < 0.3 as weak, those ≥ 0.3 and < 0.7 as moderate and those ≥ 0.7 as strong [26]. Furthermore, internal consistency was assessed for the K-BILD domains and total score with Cronbach’s alpha.

Influencing factors on HRQL were investigated via separate linear regression analyses using EQ-5D EBVS, VAS, K-BILD and the K-BILD domains as the respective outcome variables and sex, age (in years), education (basic ≤ 9 years, secondary 10-11 years, higher ≥ 12 years of schooling), employment status (full-time, part-time, unemployed), clinic location (to control also for climate differences), smoking status (current, former, never smoker), lung function parameters, disease subtype and comorbidity sum score as the independent variables. Reference categories were male, higher education, retired, study center Heidelberg, smoker and ‘other ILD’ respectively. Since the reference category for ILD subtype is more arbitrary than for the other covariables, we conducted least squares mean comparisons to detect further differences among ILD subtypes.

Given the extended recruitment period (November – April) we also investigated the potential impact of seasonal fluctuation of respiratory symptoms by including time of enrolment (winter yes/no) into our regression models. Since this more complex approach did not have a substantial additional explanatory effect, we consciously disregarded seasonal aspects within the analyses to support a straightforward interpretation.

Within a secondary analysis, we included all 14 pre-selected comorbidities to examine the influence of the distinct conditions on HRQL. Furthermore, in a sensitivity analysis we imputed the missing values except for our outcome variables (EQ-5D EBVS, VAS and K-BILD, *n* = 9). For missing categorical values we used the median of the observation (education *n* = 7, smoking status *n* = 2), and for missing continuous values the mean of the observation (DLCO % pred *n* = 16, FVC % pred *n* = 4). For the variable employment we imputed ‘full-time’ under 65 years of age and ‘retired’ above; according to the German retirement policies (*n* = 2) [27]. Additionally, we conducted the secondary analysis with imputing the lowest DLCO % pred values, in case patients with missing DLCO values were not able to take the test and thus assuming low DLCO values.

Statistical analyses were performed with SAS software (SAS Institute Inc., Cary, NC, USA, version 9.4), and *p*-values of 0.05 or less were considered statistically significant.

## Results

### Patient characteristics

Out of the 268 patients we included 229 into final analyses after excluding 39 (14.6%) with incomplete data. The excluded patients were similar to the finally included study population except for their FVC % pred (included: 70.4 vs excluded: 53.1 *p* < 0.0001) and HRQL (EQ-5D EBVS: 0.66 vs 0.59 *p* = 0.032; VAS: 61.4 vs. 49.1 *p* = 0.0005; K-BILD 53.6 vs 48.2 *p* = 0.0166).

The majority of the included patients was male (67.3%) with mean age of 63.2 (standard deviation: 12.9) and around half of the patients were retired. IPF was present in 24.0% of the patients, 22.3% presented with sarcoidosis, 11.4% HP, 9.2% ‘other IIP’, and 33.2% other ILDs (Table 1). Descriptive results stratified by center are shown in the online supplement; patients in Heidelberg were older, have more frequently basic education, were retired more often, showed higher comorbidity score and lower DLCO % pred values but showed no difference in the outcome variables (Additional file 1). The most frequent comorbidity was arterial hypertension (41.3%), followed by coronary heart disease (19.5%) and diabetes mellitus (15.9%). All other comorbidities were present in less than 10% of the study population.

**Table 1:** Baseline characteristics of the participants

Characteristic		Total sample	Excluded		p-value
*N* = 268		Mean/%	Mean/%	Missing	
		229(85.5)	39 (14.5)		
Sex *n(%)*	Male	154 (67.3)	22 (56.4)	0	0.1876
Age *Mean (SD)*		63.2 (12.9)	62.0 (13.6)	0	0.677
Education	Basic	99 (47.1)	15 (50)	9	0.6694
*n(%)*	Secondary	59 (28.1)	8 (26.7)		
	Higher	52 (24.8)	7 (23.3)		
Employment	Full-time	57 (24.9)	11 (29.7)	2	0.1379
*n(%)*	Part-time	24 (10.5)	1 (2.7)		
	Unemployed	30 (13.1)	9 (24.3)		
	Retired	118 (51.5)	16 (43.2)		
Smoking status	Current smoker	9 (3.9)	3 (8.1)	2	0.3797
*n(%)*	Former smoker	139 (60.7)	19 (51.4)		
	Never smoker	81 (35.4)	15 (40.5)		
ILD subtypes	IPF	55 (24.0)	6 (15.4)	0	0.1145
*n(%)*	Sarcoidosis	51 (22.3)	7 (18.0)		
	Hypersensitivity pneumonitis	26 (11.4)	1 (2.6)		
	Other IIPs^a^	21 (9.2)	5 (12.8)		
	Others	76 (33.2)	20 (51.3)		
DLCO% predicted *Mean (SD)*		44.2 (17.2)	44.2 (17.2)	17	0.3187
FVC % predicted *Mean (SD)*		77.4 (18.9)	53.1 (17.5)	4	<.0001
Mean number of comorbidities *Mean (SD)*		2.7 (1.8)	2.8 (2.0)	0	0.8936
EQ-5D-5L *Mean (SD)*	EBVS	0.66 (0.17)	0.6 (0.2)	6	0.0320
	VAS	61.4 (19.1)	49.1 (17.9)	5	0.0005
K-BILD	Total score	53.6 (11.7)	48.2 (10.7)	2	0.0166
*Mean(SD)*	Breathlessness and activity	41.1 (20.6)	31.6 (23.3)	0	0.0052
	Chest symptoms	64.4 (22.2)	57.4 (22.4)	1	0.0920
	Psychological impact	52.2 (13.8)	47.1 (12.1)	2	0.1014

Percentages in the excluded group show the percent of valid answers. *Abbreviations*: *SD* Standard deviation, *EQ-5D* EBVS-EQ-5D experience based value set, VAS-Visual Analog Scale, IPF-idiopathic pulmonary fibrosis, IIP-idiopathic interstitial pneumonia, EBVS-experience based value. ^a^inlcuding non-specific interstitial pneumonia, desquamative interstitial pneumonia, cryptogenetic organizing pneumonia, lymphocytic interstitial pneumonia, respiratory bronchiolitis-associated interstitial lung disease, pleuropulmonary fibroelastosis, and acute interstitial pneumonia

### ILD-specific and generic HRQL

K-BILD showed the least missing values, followed by VAS and EQ-5D EBVS with two (0.7%), five (1.87%) and six (2.24%) missing values respectively.

There was no indication for ceiling or floor effects in any outcome parameter. Regarding generic HRQL, 29 (12.7%) patients had the maximum possible score for EQ-5D EBVS, three the maximum VAS score, but no one zero. There was only one patient each within the best and worst categories for the K-BILD. K-BILD domains showed also no ceiling or floor effects (‘breathlessness and activity’ worst 5.2%, best 4.4%, ‘chest symptoms’ 0.4% vs 14.4%, ‘psychological impact’ 0.4% vs 0.4%).

**Fig. 1:**
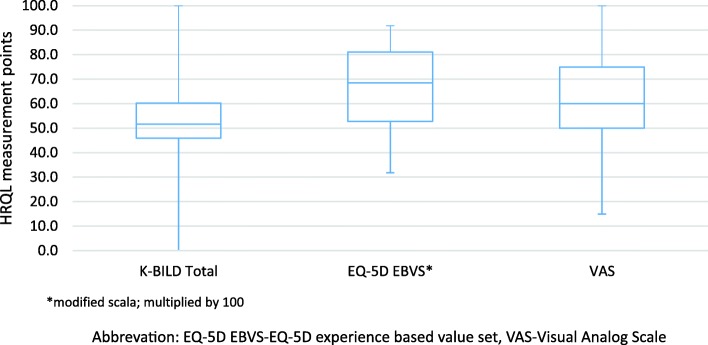
Unadjusted health-related quality of life results. Abbreviations: EQ-5D EBVS-EQ-5D experience based value set, VAS-Visual Analog Scale

Altogether, ILD-specific HRQL had lower values relative to their scale than generic HRQL (EQ-5D EBVS: 0.66 and VAS: 61.4 vs K-BILD: 53.6) (Fig. 1) with the highest impairments occurring in the ‘breathlessness and activity’ domain (unadjusted mean scores: 41.1 vs. 52.2 ‘psychological impact’ and 64.4 ‘chest symptoms’).

### Correlation of HRQL assessment tools and internal consistency

K-BILD total score correlated strongly with the EQ-5D EBVS (0.71), but only moderately with the VAS (0.55). All instruments had weak or moderate correlations with lung function parameters and comorbidity burden (Table 2). The K-BILD domains showed stronger correlations to the EQ-5D EBVS than to the VAS. Looking at correlations between the K-BILD and the EQ-5D dimensions, the strongest correlation was found for the ‘breathlessness and activity’ (K-BILD) with ‘usual activities’ (EQ-5D) (− 0.69, *p* < 0.00001) and ‘mobility’ (EQ-5D) (− 0.65, *p* < 0.0001). However, further correlations were moderate at best (Table 3).

**Table 2:** The correlation between health status and lung function

	EQ-5D EBVS	VAS	K-BILD Total	K-BILD Breath	K-BILD Chest	K-BILD Psych	FVC % predicted	DLCO % predicted	Comorbidity sum score
EQ-5D EBVS	1								
VAS	0.58 (<.0001)	1							
K-BILD Total	0.71 (<.0001)	0.55 (<.0001)	1						
K-BILD Breath	0.71 (<.0001)	0.58 (<.0001)	0.86 (<.0001)	1					
K-BILD Chest	0.60 (<.0001)	0.47 (<.0001)	0.78 (<.0001)	0.61 (<.0001)	1				
K-BILD Psych	0.60 (<.0001)	0.49 (<.0001)	0.93 (<.0001)	0.67 (<.0001)	0.69 (<.0001)	1			
FVC % predicted	0.30 (<.0001)	0.21 (0.0013)	0.29 (<.0001)	0.36 (<.0001)	0.22 (0.0006)	0.24 (0.0002)	1		
DLCO % predicted	0.17 (0.0106)	0.14 (0.0409)	0.27 (<.0001)	0.35 (<.0001)	0.12 (0.0821)	0.22 (0.001)	0.47 (<.0001)	1	
Comorbidity sum score	−0.26 (<.0001)	− 0.25 (0.0002)	− 0.21 (0.0012)	− 0.28 (<.0001)	−0.16 (0.0173)	− 0.16 (0.0174)	− 0.09 (0.1569)	− 0.26 (<.0001)	1

*Abbreviations*; K-BILD Breath- K-BILD Breathlessness and activity, K-BILD Chest- K-BILD Chest symptoms, K-BILD Psych- K-BILD Psychological Impact, FVC % pred –Forced vital capacity % predicted, DLCO % predicted- Carbon monoxide diffusing capacity % predicted. In brackets we reported *p*-values. We considered correlations < 0.3 as weak, ≥0.3 and < 0.7 as moderate and ≥ 0.7 as strong

**Table 3:** Relationship between the different HRQL domains

	EQ-5D Mobility	EQ-5D Self-care	EQ-5D Usual activities	EQ-5D Pain/ discomfort	EQ-5D Anxiety/ depression
EQ-5D Mobility	1				
EQ-5D Self-care	0.51 (<.0001)	1			
EQ-5D Usual activities	0.65 (<.0001)	0.54 (<.0001)	1		
EQ-5D Pain/ discomfort	0.40 (<.0001)	0.29 (<.0001)	0.49 (<.0001)	1	
EQ-5D Anxiety/ depression	0.39 (<.0001)	0.26 (<.0001)	0.44 (<.0001)	0.24 (0003)	1
K-BILD Breath	−0.65 (<.0001)	− 0.48 (<.0001)	− 0.69 (<.0001)	− 0.45 (<.0001)	− 0.38 (<.0001)
K-BILD Chest	− 0.49 (<.0001)	− 0.31 (<.0001)	−0.52 (<.0001)	− 0.45 (<.0001)	−0.35 (<.0001)
K-BILD Psych.	−0.47 (<.0001)	−0.33 (<.0001)	− 0.53 (<.0001)	−0.39 (<.0001)	− 0.51 (<.0001)

*Abbreviations*; K-BILD Breath- K-BILD Breathlessness and activity, K-BILD Chest- K-BILD Chest symptoms, K-BILD Psych- K-BILD Psychological Impact. In brackets, we reported *p*-values. We considered correlations < 0.3 as weak, ≥0.3 and < 0.7 as moderate and ≥ 0.7 as strong

The K-BILD total score showed the highest internal consistency with Cronbach’s alpha of 0.89, followed by the ‘breathlessness and activity’ domain, ‘chest symptoms’ and psychological impact with values 0.87, 0.74 and 0.73 respectively.

### Impact factors on ILD-specific and generic HRQL

In the primary analysis, the strongest influencing factor for all of the HRQL measures and their domains was FVC % pred (Table 4). Older age, higher education and working full time were associated with higher EQ-5D EBVS but did not significantly influence K-BILD or VAS. Patients classified as other ILDs had worse HRQL compared to IPF patients (measured with EQ-5D EBVS, VAS and ‘breathlessness and activity’ domain) or compared to ‘other IIP’ patients (measured with EQ-5D EBVS and with ‘psychological impact’ domain) (Table 4). Least square mean comparisons between the remaining groups showed in two cases a difference in the primary analysis. Regarding EQ-5D EBVS sarcoidosis patients had lower values than patients did in the ‘other IIP’ group (− 0.098 *p* = 0.0278). Moreover, they were significantly more impaired in the ‘breathlessness and activity’ domain of K-BILD compared to IPF patients (− 12.19 *p* = 0.0089) (Additional file 2).

**Table 4:** Results of regression analyses for the primary analysis (with comorbidity score)

				K-BILD			
		EQ-5D EBVS	VAS	Total	Breathlessness and activity	Chest symptoms	Psychological impact
	Parameter	Estimate	Estimate	Estimate	Estimate	Estimate	Estimate
Sex *(Ref = Male)*	Female	−0.016	2.63	−0.99	−1.39	− 0.90	− 1.49
Age		0.003**	−0.02	0.12	0.06	0.22	0.13
Education	Basic	−0.05*	−5.44	−1.85	−4.47	− 1.66	− 0.84
*(Ref = higher)*	Secondary	0.03	−0.65	0.24	0.16	2.86	0.14
Employment	Full-time	0.11**	6.05	3.78	5.85	7.19	2.31
*(Ref = retired)*	Part-time	0.07	7.9	3.39	7.26	2.13	3.27
	Not employed	−0.02	−3.38	−0.23	−1.03	−3.49	−0.19
Clinic *(Ref = GH)*	Heidelberg	0.03	1.86	2.28	3.61	4.25	3.05
Smoking status *(Ref = smoker)*	Never smoker	0.03	0.75	6.00	3.01	6.45	9.53*
	Former Smoker	−0.02	0.45	−2.69	−4.52	−4.71	−2.59
FVC % pred		0.002***	0.19*	0.15**	0.28***	0.27**	0.16**
DLCO % pred		−0.0004	−0.06	0.07	0.20*	−0.004	0.06
Disease Subtype *(Ref = other)*	IPF	0.07*	8.39*	3.51	7.85*	3.62	2.71
	Sarcoidosis	−0.001	−0.49	− 0.90	−4.34	− 5.71	0.67
	HP	0.02	0.73	3.33	3.29	4.89	4.43
	Other IIPs^1^	0.09*	8.78	4.70	5.48	1.16	7.29*
Comorbidity sum score		−0.03***	−2.72**	−1.51**	−3.06***	− 2.68**	− 1.44*

Values depicted are the beta estimates of regression coefficients. *Abbreviations*: IPF-idiopathic pulmonary fibrosis, HP-Hypersensitivity pnemonitis, GH-Großhansdorf. ^1^including: non-specific interstitial pneumonia, desquamative interstitial pneumonia, cryptogenetic organizing pneumonia, lymphocytic interstitial pneumonia,respiratory bronchiolitis-associated interstitial lung disease, pleuropulmonary fibroelastosis, and acute interstitial pneumonia. *p ≤ 0.05, **p ≤ 0.01, ***p ≤ 0.001

The secondary analysis revealed two comorbidities with a significant influence; arterial hypertension was associated with a lower EQ-5D EBVS (− 0.05 *p* = 0.0441) and with a lower score in the ‘breathlessness and activity’ domain (− 6.85 *p* = 0.0173) (Table 5). Additionally, depression had a strong negative association with the ‘chest symptoms’ domain (− 17.04 *P* = 0.0029).

**Table 5:** Results of regression analyses for the comorbidities in the secondary analysis

			K-BILD			
	EQ-5D EBVS	VAS	Total	Breathlessness	Chest symptoms	Psychological impact
	Est.	Est.	Est.	Est.	Est.	Est.
Comorbidities						
Pulmonary hypertension	−0.02	2.99	2.43	1.74	5.71	3.33
Arterial hypertension	−0.05*	−2.96	−2.75	−6.85*	−1.04	− 2.08
Coronary heart disease	−0.02	−4.70	−2.06	− 2.90	−5.97	− 2.51
Congestive heart failure	0.05	−4.57	2.12	3.22	3.81	1.81
Other CVD	0.02	10.96	4.07	0.98	16.60	5.34
Diabetes mellitus	−0.03	−0.96	−1.89	0.35	−3.94	−1.91
Emphysema/COPD	−0.06	− 3.02	− 3.18	−6.03	−9.11	−2.38
Lung cancer	0	0	0	0	0	0
Depression	−0.08	−5.27	−5.36	−5.69	−17.04**	−5.87
GERD	0.04	−6.87	−0.86	−1.72	−4.39	−2.62
Renal failure	0.08	2.83	3.79	8.87	6.28	1.82
OSAS	−0.05	−6.11	−1.71	−3.66	−8.22	−1.44
Thromboembolism	−0.11	−2.82	−2.27	0.31	−9.70	−4.93
Malignant tumor	−0.01	−13.66	−0.68	3.60	−13.06	−1.28

Values depicted are the beta estimates of regression coefficients,all adjusted for age, sex, education, employment,clinic location, smoking status, FVC % pred, DLCO % pred, disease suptype. *Abbreviations* Est-estimates, *IPF* idiopathic pulmonary fibrosis, HP: hypersensitivity pnemonitis ^1^including: non-specific interstitial pneumonia, desquamative interstitial pneumonia, cryptogenetic organizing pneumonia, lymphocytic interstitial pneumonia,respiratory bronchiolitis associated interstitial lung disease, pleuropulmonary fibroelastosis, and acute interstitial pneumonia. *p ≤ 0.05, **p ≤ 0.01, ***p ≤ 0.001

Within the sensitivity analyses (*n* = 257) results changed only marginally in terms of effect sizes as well as in terms of significant levels. Altogether, the results of the main analyses were mirrored almost perfectly without any noteworthy exceptions.

## Discussion

Here, we provide first comprehensive data on HRQL in real life settings in Germany of a large ILD cohort and compared a ILD-specific HRQL questionnaire (K-BILD) with the generic EQ-5D-5L in order to examine its suitability to measure HRQL of ILD patients in a German setting.

In summary our results show, that K-BILD is well accepted among patients (low number of missing values) and its results in Germany are comparable to those of other studies [9, 10, 28], thus supporting the use of K-BILD in Germany. Additionally, further analysis showed the EQ-5D-5L to have properties similar to the K-BILD and hence allowing its use in the ILD disease group, and open up comparability of ILD disease burden in terms of HRQL to that of other diseases as well as to HRQL in the general population.

Both instruments lack floor and ceiling effects, indicating that they should be able to detect changes in the patients HRQL over time, which is important for further clinical research. Accordingly, the implementation of these tools could promote better understanding of the impairments in ILD in different countries, among different study populations and the HRQL development throughout time.

Our study was the first applying the K-BILD in a German observational study and comparisons to international evidence need to be interpreted keeping different healthcare environment and patient preferences in mind. Three studies from different European contries reported comparable mean K-BILD scores as our study [9, 10, 28] Additionally, in line with our findings, the ILD patients of the Wapenaar study had the greatest impairment in the ‘breathlessness and activity’ domain, followed by ‘psychological impact’ and ‘chest symptoms’. This strongly supports the international transferability and applicability of K-BILD. Despite the high concordance of K-BILD scores cross-nationally and the lack of any other ILD-specific questionnaire, the tool is sparsely used. Our results suggest that a more frequent use would be beneficial.

K-BILD showed strong correlation with the EQ-5D EBVS, suggesting that it measures similar aspects. At the same time, K-BILD showed stronger correlations with lung function parameters than EQ-5D-5L. This emphasizes the assumption that K-BILD may be more suitable to detect the impairments originating from ILD. Since EQ-5D-5L might not be sensitive enough in case of disease-specific conditions [29], it is especially important to find a valid instrument measuring disease-specific burden to foster a more targeted patient-centered ILD management.

The trend of lower correlations between the HRQL domains highlights once more the difference between generic and disease-specific questionnaires, but the high correlation of the K-BILD and EQ-5D EBVS allows us to still assume a good overall picture about the HRQL. Our results show in almost all cases slightly lower but still comparable correlation coefficients (EQ-5D, VAS vs K-BILD and its domains) than from the language validation from Wapenaar et al. [10]. Furthermore, reliability measured with Chronbach’s alpha showed comparable results (good or moderate) as in Patel et al. [9] and in Wapenaar et al. [10], proving the consistency of K-BILD.

As expected EQ-5D EBVS reacted more sensitive to sociodemographic factors (age, sex) and socioeconomic status (employment) than K-BILD, since generic instruments are known to implicitly cover generic health aspects more comprehensively than disease-specific ones. The unexpected results of the association of higher age with higher HRQL could occur because older people have lower expectations regarding HRQL or maybe because of further undetected covariables, for which are not accounted in this setting. The disease-specific K-BILD showed in the domains ‘breathlessness and activity’ and ‘chest symptoms’ a high sensitivity for the lung function value FVC % pred. These findings are in contradiction with Coelho et al., who did not find any association by applying Medical Outcomes Study Short Form 36 -item questionnaire and SGRQ [8]. This could be due to the lower number of patients or due to use of a different HRQL assessment tool in their study. Worth mentioning, HRQL assessment tools are meant to quantify important aspects of the patients’ subjective well-being than objective clinical outcomes, therefore low correlations between lung function values and HRQL seem to be tolerable.

Results from King et al. as well as from Kreuter et al. suggest that increasing comorbidity burden is associated with increased morbidity and mortality [30, 31]. Apart from these hard outcomes, the number of comorbid conditions has apparently also a detrimental effect on self-rated HRQL of ILD patients. Therefore, improved comorbidity management in ILD patients might not only reduce the mortality risk itself but also contribute to improve HRQL. In this regard, the generic EQ-5D EBVS and VAS react stronger to comorbidity than K-BILD. The reason could be the high severity of the ILD and thus overpowering other comorbidities in the psychological aspects. Even though our study population differed regarding their HRQL from the excluded patients (showed significantly lower values), the sensitivity analysis confirmed our primary results.

The secondary analysis revealed that, despite the significant association between comorbidity burden and HRQL, only a few distinct comorbidities seem decisive for HRQL. Depression was negatively associated with HRQL in the ‘chest symptoms’ domain. This could reflect the high burden of the underlying ILD or could be due to the general association between chest pain and depression independent of ILD [32]. The lack of association between HRQL and other comorbidities could be due to the low number of patients for the distinct comorbidities. Furthermore, these results suggest the additivity of the effects of the comorbidities on HRQL.

There is no clinical evidence for the measured higher impairment of sarcoidosis patients in comparison with other subtypes yet; further research is needed in this regard. A possible explanation could be that compared to diseases restricted to the lungs, e.g. IPF or HP, sarcoidosis is a systemic disease with systemic consequences.

Our findings have to be interpreted under some caveats. As with any observational cross-sectional study, our results show associations but causality cannot be tested. Controlling for confounders was the best strategy to address this issue but some important confounder might have been overlooked. Furthermore, this study was voluntary, therefore selection bias cannot be ruled out. Given the high accordance of our findings to international evidence on K-BILD, we consider selection bias to be of minor importance. Additionally, the results may not be generalizable to populations outside Germany.

The crucial strength of our study is that we applied K-BILD for the first time in a German setting in a comparatively large patient cohort consisting of individuals with various subtypes located on quite distant geographic location. This enables general conclusions on the suitability of K-BILD as the disease-specific HRQL measurement of choice.

## Conclusion

In conclusion, K-BILD and EQ-5D revealed similar HRQL trends and were sensitive to the same disease-related factors. K-BILD reacted more sensitively to ILD-specific aspects of HRQL rendering it a valuable complementary measure to the generic EQ-5D-5L. Therefore, we propose that K-BILD should be implemented as standard tool in clinical practice.

## Additional files

Additional file 1: Baseline characteristics stratified by clinic. (DOCX 17 kb)

Additional file 2: Least squares means comparison among disease subtypes for the primary analysis. (DOCX 17 kb)

## Abbreviations

COPD: Chronic obstructive pulmonary disease
DLCO % pred: Diffusing capacity of the lungs for carbon monoxide percent predicted
EQ-5D EBVS: EQ-5D experience based value set
EQ-5D-5L: EuroQol 5 dimensions
FVC % pred: Forced vital capacity percent predicted
HP: Hypersensitivity pneumonitis
HRQL: Health-related quality of life
IIP: Idiopathic interstitial pneumonia
ILD: Interstitial lung diseases
IPF: Idiopathic pulmonary fibrosis
K-BILD: King’s Brief Interstitial Lung Disease Questionnaire
SGRQ: St George’s Respiratory Questionnaire
VAS: Visual Analog Scale
