# Supplementary material for: Quality of life assessment in interstitial lung diseases:a comparison of the disease-specific K-BILD with the generic EQ-5D-5L
Source: Respir Res. 2018 May 25;19:101. doi: 10.1186/s12931-018-0808-x (PMC5970441; doi:10.1186/s12931-018-0808-x)
Supplement: Supplementary file 1 — Baseline characteristics stratified by clinic. (DOCX 17 kb) [file 12931_2018_808_MOESM1_ESM.docx]

| **Additional file 1: Baseline characteristics stratified by clinic** | | | | |  |
| --- | --- | --- | --- | --- | --- |
| Characteristic |  | Total sample | Großhansdorf | Heidelberg | p-value |
|  |  |  |  |  |  |
| N (%) |  | 229 (100) | 68 (29.7) | 161 (70.3) |  |
| Sex | Male n(%) | 154 (67.3) | 44 (64.7) | 110 (68.3) | 0.5941 |
| Age Mean (SD) |  | 63.21 (12.9) | 57 (13.9) | 65.8 (11.6) | <.0001 |
| Education | Basic | 99 (47.1) | 22 (33.9) | 77 (53.1) | 0.047 |
| *n(%)* | Secondary | 59 (28.1) | 24 (36.9) | 35 (24.1) |  |
|  | Higher | 52 (24.8) | 19 (29.2) | 33 (22.8) |  |
| Employment | Full-time | 57 (24.9) | 24 (35.3) | 33 (20.5) | 0.0269 |
| *n(%)* | Part-time | 24 (10.5) | 9 (13.2) | 15 (9.3) |  |
|  | Unemployed | 30 (13.1) | 10 (14.7) | 20 (12.4) |  |
|  | Retired | 118 (51.5) | 25 (36.8) | 93 (57.8) |  |
| Smoking status | Current smoker | 9 (3.9) | 3 (4.4) | 6 (3.7) | 0.7946 |
| *n(%)* | Former smoker | 139 (60.7) | 39 (57.4) | 100 (62.1) |  |
|  | Never smoker | 81 (35.4) | 26 (38.2) | 55 (34.2) |  |
| ILD subtypes | IPF | 55 (24.0) | 6 (8.8) | 49 (30.4) | <.0001 |
| *n(%)* | Sarcoidosis | 51 (22.3) | 31 (45.6) | 20 (12.4) |  |
|  | Hypersensitivity pneumonitis | 26 (11.35) | 8 (11.76) | 18 (11.18) |  |
|  | Other IIPs¹ | 21 (9.17) | 3 (4.41) | 18 (11.18) |  |
|  | Others | 76 (33.19) | 20 (29.41) | 56 (34.78) |  |
| DLCO% predicted  *Mean (SD)* |  | 44.2 (17.2) | 49.9 (19.4) | 41.9 (15.7) | 0.0031 |
| FVC % predicted  *Mean (SD)* |  | 77.4 (18.9) | 73.2 (17.9) | 69.3 (19.2) | 0.534 |
| Mean number of comorbidities *Mean (SD)* |  | 2.7 (1.8) | 1.7 (1.5) | 3.1 (1.7) | <.0001 |
| EQ-5D-5L  *Mean (SD)* | EBVS | 0.66 (0.17) | 0.66 (0.17) | 0.66 (0.17) | 0.924 |
|  | VAS | 61.4 (19.1) | 62.8 (18.3) | 60.8 (19.5) | 0.4915 |
| K-BILD | Total score | 53.6 (11.7) | 53.4 (11.5) | 53.6 (11.7) | 0.9071 |
| *Mean(SD)* | Breathlessness and activity | 41.1 (20.6) | 42.3 (21.5) | 40.6 (20.2) | 0.579 |
|  | Chest symptoms | 64.4 (22.2) | 62.9 (20.8) | 65.0 (22.9) | 0.5238 |
|  | Psychological impact | 52.2 (13.8) | 51.5 (12.6) | 52.5 (14.3) | 0.5994 |
| Abbreviations: SD-Standard deviation, EQ-5D EBVS-EQ-5D experience based value set, VAS-Visual Analog Scale, IPF-idiopathic pulmonary fibrosis, IIP-idiopathic interstitial pneumonia, EBVS-expreience based value. ¹inlcuding non-specific interstitial pneumonia, desquamative interstitial pneumonia, cryptogenetic organizing pneumonia, lymphocytic interstitial pneumonia,respiratory bronchiolitis-associated interstitial lung disese, pleuropulmonary fibroelastosis, and acute interstitial pneumonia. | | | | |  |
