# Supplementary material for: Quality of life assessment in interstitial lung diseases:a comparison of the disease-specific K-BILD with the generic EQ-5D-5L
Source: Respir Res. 2018 May 25;19:101. doi: 10.1186/s12931-018-0808-x (PMC5970441; doi:10.1186/s12931-018-0808-x)
Supplement: Supplementary file 2 — Least squares means comparison among disease subtypes for the primary analysis. (DOCX 17 kb) [file 12931_2018_808_MOESM2_ESM.docx]

| **Additional file 2: Least squares means comparison among disease subtypes for the primary analysis** | | | | | | | | | | | | |
| --- | --- | --- | --- | --- | --- | --- | --- | --- | --- | --- | --- | --- |
|  |  | |  | | **K-BILD** | | | | | | | |
|  | **EQ-5D EBVS** | | **VAS** | | **Total** | | **Breathlessness and activity** | | **Chest symptoms** | | **Psychological impact** | |
|  | **LS Mean** | **SE** | **LS Mean** | **SE** | **LS Mean** | **SE** | **LS Mean** | **SE** | **LS Mean** | **SE** | **LS Mean** | **SE** |
| **IPF** | 0.73 | 0.03 | 68.33 | 3.53 | 58.79 | 2.10 | 50.70 | 3.54 | 71.23 | 4.19 | 57.03 | 2.57 |
| **SAR** | 0.66 | 0.03 | 59.44 | 3.73 | 54.37 | 2.22 | 38.51 | 3.74 | 61.90 | 4.43 | 54.99 | 2.71 |
| **HP** | 0.69 | 0.04 | 60.67 | 4.35 | 58.61 | 2.58 | 46.14 | 4.36 | 72.51 | 5.16 | 58.76 | 3.16 |
| **Other IIP** | 0.76 | 0.04 | 68.71 | 4.59 | 59.98 | 2.73 | 48.33 | 4.60 | 68.77 | 5.44 | 61.62 | 3.33 |
| **Other** | 0.66 | 0.02 | 59.94 | 2.95 | 55.28 | 1.75 | 42.85 | 2.95 | 67.61 | 3.49 | 54.32 | 2.14 |
|  |  |  |  |  |  |  |  |  |  |  |  |  |
|  | **Diff. in LS-Means** | **p-value** | **Diff. in LS-Means** | **p-value** | **Diff. in LS-Means** | **p-value** | **Diff. in LS-Means** | **p-value** | **Diff. in LS-Means** | **p-value** | **Diff. in LS-Means** | **p-value** |
| **SAR vs Other IIP** | -0.10 | 0.0278 | -9.27 | 0.0843 | -5.60 | 0.0791 | -9.82 | 0.0682 | -6.86 | 0.2804 | -6.63 | 0.0892 |
| **SAR vs HP** | -0.03 | 0.5339 | -1.23 | 0.8014 | -4.23 | 0.1467 | -7.63 | 0.121 | -10.60 | 0.0689 | -3.77 | 0.2899 |
| **SAR vs IPF** | -0.07 | 0.0534 | -8.89 | 0.055 | -4.42 | 0.108 | -12.19 | 0.0089 | -9.33 | 0.0892 | -2.04 | 0.5418 |
| **SAR vs Other** | 0.00 | 0.9714 | -0.50 | 0.9014 | -0.90 | 0.7048 | -4.34 | 0.2814 | -5.71 | 0.2316 | 0.67 | 0.8194 |
| **Other IIP vs HP** | 0.07 | 0.1086 | 8.04 | 0.1424 | 1.37 | 0.6726 | 2.19 | 0.6891 | -3.74 | 0.5643 | 2.86 | 0.4713 |
| **Other IIP vs IPF** | 0.02 | 0.5492 | 0.39 | 0.9366 | 1.19 | 0.6795 | -2.37 | 0.6249 | -2.47 | 0.6675 | 4.59 | 0.1929 |
| **Other IIP vs Other** | 0.10 | 0.0118 | 8.78 | 0.0582 | 4.70 | 0.0875 | 5.48 | 0.2367 | 1.16 | 0.8328 | 7.30 | 0.0304 |
| **HP vs IPF** | -0.05 | 0.203 | -7.66 | 0.099 | -0.18 | 0.9464 | -4.56 | 0.3254 | 1.27 | 0.8164 | 1.72 | 0.6081 |
| **HP vs Other** | 0.02 | 0.4995 | 0.73 | 0.8644 | 3.33 | 0.1934 | 3.29 | 0.4455 | 4.90 | 0.3373 | 4.43 | 0.1564 |
| **IPF vs Other** | 0.07 | 0.0115 | 8.39 | 0.0159 | 3.51 | 0.0882 | 7.85 | 0.0242 | 3.62 | 0.3774 | 2.71 | 0.2808 |
| Abbreviations: LS-Mean- Least squares means, SE- standard error, Diff-difference, IPF-idiopathic pulmonary fibrosis, SAR-sarcoidosis, HP-Hypersensitivity pneumonitis, IIP- idiopathic interstitial pneumonia. Every reported LS-Mean has a p-value of <0.0001 | | | | | | | | | | | | |
